# Height Variation Depending on the Source of Antenna Phase Centre Corrections: LEIAR25.R3 Case Study

**DOI:** 10.3390/s19184010

**Published:** 2019-09-17

**Authors:** Andrzej Araszkiewicz, Damian Kiliszek, Anna Podkowa

**Affiliations:** 1 Faculty of Civil Engineering and Geodesy, Military University of Technology, gen. S. Kaliskiego 2, 00-908 Warsaw, Poland; damian.kiliszek@wat.edu.pl; 2 Faculty of Geodesy and Cartography, Warsaw University of Technology, Pl. Politechniki 1, 00-661 Warsaw, Poland; amj.podkowa@op.pl

**Keywords:** GPS antenna, phase center variations, height estimates, minimum observation angle

## Abstract

In this study, we compared two sets of antenna phase center corrections for groups of the same type of antenna mounted at the continuously operating global navigation satellite system (GNSS) reference stations. The first set involved type mean models provided by the International GNSS Service (release igs08), while the second set involved individual models developed by Geo++. Our goal was to check which set gave better results in the case of height estimation. The paper presents the differences between models and their impact on resulting height. Analyses showed that, in terms of the stability of the determined height, as well as its variability caused by increasing the facade mask, both models gave very similar results. Finally, we present a method for how to estimate the impact of differences in phase center corrections on height changes.

## 1. Introduction

Global navigation satellite systems are applicable in many areas of life and are probably the most popular method for positioning. Their continuous development introduced new methods and improved algorithms [1,2,3], allowing the determination of the accuracy of estimated position [4] and testing the possibilities of using three frequencies [1,5]. This development increased the accuracy of the positioning with shorter sessions. GNSSs are used for measurements wherever a stable and precise reference frame is required, such as landslides [6], and subsidence or secular tectonic movements [7]. They also have wide applications in Earth sciences, including in atmosphere research [8,9] or determining the position of the Earth’s mass center [10]. All these works require the most accurate determination of parameters, which is particularly important for the integration of GNSS measurements with other remote sensors [11] or direct measurements [12]. To be able to provide millimeter accuracy, it is necessary to take into account the impact of the receiving system, especially the antenna.

The purpose of GNSS antennas is to convert the energy of the electromagnetic wave to electric current, which is then processed by the receiver. The currently constructed antennas are adapted to receive the wave at individual frequencies corresponding to the frequencies of individual GNSS systems. Recent systems enable reception waves on all available frequencies, e.g., L1/L2/L5 for GPS, G1/G2/G3 for GLONASS, E1/E5a/E5b/E5/E6 for Galileo, and B1/B3/B1C/B2a/B2b for BDS [13,14]. However, the most popular ones are those receiving the signal from two frequencies, and, because of their price and ensuring sufficient accuracy for many years, they will dominate the market. In theory, the best antenna is the one with isotropic distribution of radiation intensity; however, for technical reasons, it is impossible to construct. The most popular antennas are omni-directional antennas with the same radio power in the azimuthal direction and varying power for elevation angles. In practice, the actual antenna differs slightly from this ideal. For this reason, the position of the phase center is not constant and differs from the direction of the electromagnetic wave. It is also different for different frequencies. For this reason, in precise positioning, it is necessary to model the location of the phase center and its variability for both receiving and transmitting antennas. It is widely accepted to determine the location of the phase center relative to the reference point of the antenna (ARP). Antenna models are inextricably related through two definitions: the phase center offset (PCO), which expresses the mean position of the phase center from all directions, and the phase center variation (PCV), which is the deviation from the mean position in a given direction. Together, they represent the phase center correction (PCC), which is modeled in the processing for each direction on a given frequency. For the general concept, see References [15,16].

PCC values are created during the calibration process. There are basically two kinds of calibrations. The first is relative, where PCCs are designated in relation to the reference antenna [17]. The second is an absolute model, where PCCs are independent values. Currently, relative models are not recommended by the International GNSS Service (IGS) for use in analysis. They were replaced by absolute models, which are generally more accurate and freer of errors than the reference antenna itself [17]. Currently, two absolute calibration approaches are used. The first of them (used, e.g., by Geo++^®^ GmbH [18], Leibniz University Hannover, Institut für Erdmessung [19], or Wuhan University [20]) uses a special robot for this purpose. In this case, the estimated values are based on the real GNSS signal. The second method (used, e.g., by University of Bonn, Institute of Geodesy and Geoinformation [21]) is based on anechoic chambers, and PCC values come from a given simulated frequency. Each antenna is different; therefore, the result of calibration is an individual PCC table dedicated to one specific antenna. Specific examples of absolute models are the type mean models provided by the IGS [22]. They are based on many calibration tables available for the same type of antenna. The resulting mean models are IGS products and are recommended for use in IGS analyses. As a result, IGS type mean models can be used for antennas that do not have individual models available. This indicates that various sources of PCCs are available for individual antennas, whether individual or type mean, which can potentially be used in analysis. For example, for the International Association of Geodesy Regional Reference Frame sub-commission for Europe, EUREF [23], individual models should be used whenever they are available [24]. Only in their absence is the use of IGS type mean models recommended. However, this is not a general standard; these are only recommendations for EUREF Permanent GNSS Network (EPN) Analysis Centers.

All measurement calibrations are burdened with errors. In both cases, calibration tables do not have an estimated accuracy. Their reliability can be determined by comparing available models of the same antennas developed by different institutions and by different methods. In Reference [25], the authors showed that, in general, the agreement between different calibrations is on a millimeter level. The impact of using different antenna models on the estimated coordinates was discussed in Reference [26]. In Reference [27], the authors showed the potential impact on frame realization at the regional level. Analyses were also carried out on the effect of mixing PCC sources on tropospheric parameters [28,29], which caused discrepancies up to the millimeter level. In addition, in Reference [30], the authors presented a methodology to investigate and validate the calibration tables. Based on the results, they found significant differences between individual calibration sets, which allowed estimating coordinate corrections and finally removing systematic biases and achieving a better agreement with truth values. For this purpose, they designed a special polygon on which selected antennas were tested.

In our analyses, we decided to find a method that would allow estimating the impact of used PCCs without the need for additional measurements or special construction. We wanted to check if this was possible on the basis of already existing observations. We focused only on the height estimates, as they are most affected. We compared the available PCC tables for selected EPN stations, and we checked which of them gave the most stable results. We also made an attempt to show how differences in PCCs translate to differences in height.

## 2. Methodology

For our analysis, GPS observations collected at 24 selected stations belonging to EPN were used in this study. The analysis was performed on full-year 2015 data and focused on one type of antenna, the most popular of the ground antennas mounted at that time in EPN, the GNSS Choke Ring Antenna Type AR25 from Leica (IGS code: LEIAR25.R3) with Radome LEIT [31].

**Figure 1:**
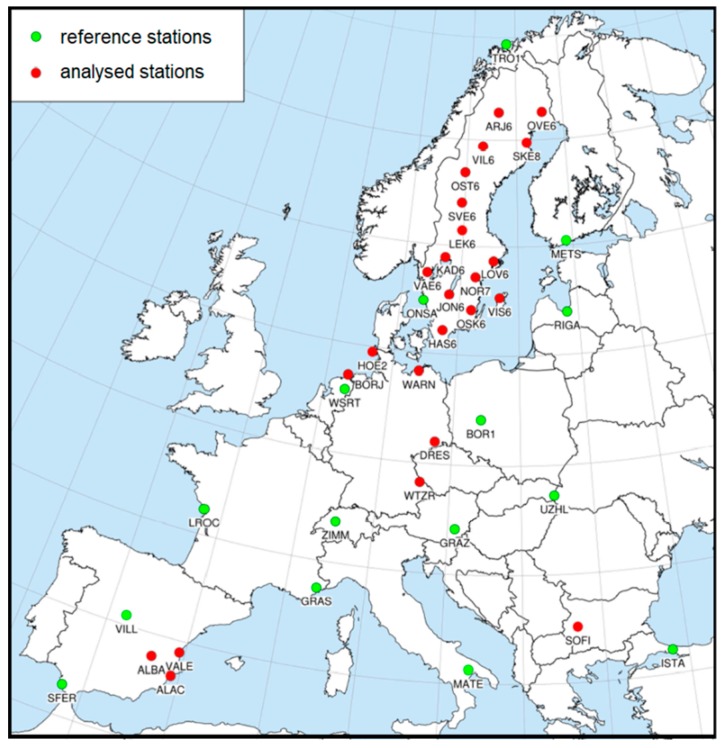
Map of the stations included in this research. Red points indicate stations equipped with analyzed antennas, while green points indicate the reference stations used for network alignment.

All GPS data were processed in the Gamit/Globk v.10.60 software [32]. The processing strategy followed EPN Guidelines and corresponded to the contribution by the Military University of Technology to the EPN Repro2 project [26]. To correct the phase observations, two ground antenna phase center correction sets were used. The first one, igs08.atx, contains corrections for types of GNSS antennas recognized by IGS, where the values are means from several calibrations [22]. The second one, epnc.atx [23], contains corrections for individual antennas derived from individual calibrations made by Geo++ GmbH and collected by EPN Central Bureau (Belgium). The network alignment to the IGb08 frame was made using 14 additional stations (Figure 1) using no-net-translation minimum constraint conditions. The processing strategy was the same for the reference stations and was manipulated for the remaining stations. For each calibration table, nine solutions with different elevation masks were prepared (Table 1).

**Figure 2:**
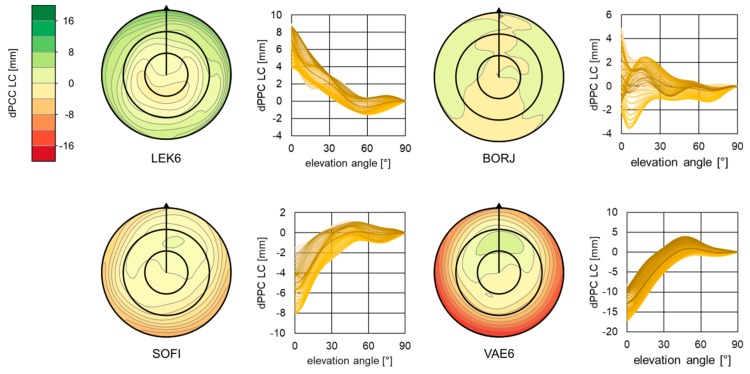
Differences in the phase center corrections (PCCs) for “ionosphere-free” linear combination for the LEIAR25.R3 LEIT antenna at selected EUREF permanent global navigation satellite system (GNSS) network (EPN) stations.

Before the investigation of height estimates, the PCC values for individual antennas were compared. The comparison procedure is explained in detail in References [19,33]. Firstly, the PCOs from epnc.atx were transferred to a common PCO with igs_08.atx according to Reference [19]. Then, independently for both GPS frequencies, PCVs were subtracted as EPN − IGS. Differences for L1 and L2 obtained in this way were used to calculate the final difference in PCCs (dPCCs) for “ionosphere-free” linear combination (LC). For selected stations, resulting dPCCs are presented in Figure 2.

As it was expected, the greatest differences between compared correction sets occurred at low elevations, where they were close to 20 mm (Figure 2). This came from the original patterns (e.g., igs_08.atx), which adopted a PCV(ϕ,90°)=0 mm condition and, therefore, showed higher values at lowest elevations. Corrections at the lowest elevation also had the highest standard deviations [18]. The greatest differences between compared PCCs were for station VAE6, where the total RMS for LC dPCC was 4.9 mm. As already shown in Reference [27] for this station, the change of PCC from IGS type mean to individual calibration may affect the estimated height by over 15 mm. However, this was an exception. The mean RMS for all LC dPCCs was close to 2.0 mm (Table A1, Appendix A). The highest values occurred for the antennas at new Swedish stations. In general, dPCCs show an azimuthal symmetry with a lower consistency occurring at lower elevations (Table A2, Appendix A). Among all investigated stations/antennas, the mean azimuthal RMS, calculated as mean RMS from all RMSs for each elevation, was 1.12 mm (minimum = 0.02; maximum = 3.08 mm), whereas the elevational RMS, calculated as mean RMS from all RMSs for each azimuth, was 1.41 mm (minimum = 0.19 mm; maximum = 5.70 mm). This common feature of analyzed dPCCs explains partly why the change between type mean and individual calibrations mostly impacts estimated heights. Analyzing the elevational discrepancies in dPCCs, one can distinguish the following two groups of patterns:

- The first one, where the differences are very small and can be considered as insignificant (e.g., stations BORJ, NOR7).
- The second one, where differences are positive (e.g., LEK6) or negative (e.g., OST6, VAE6) at low elevations and are gradually closer to 0 (e.g., OST6, VAE6).

Such patterns have a direct impact on the results, as described in the next section.

## 3. Results

**Figure 3:**
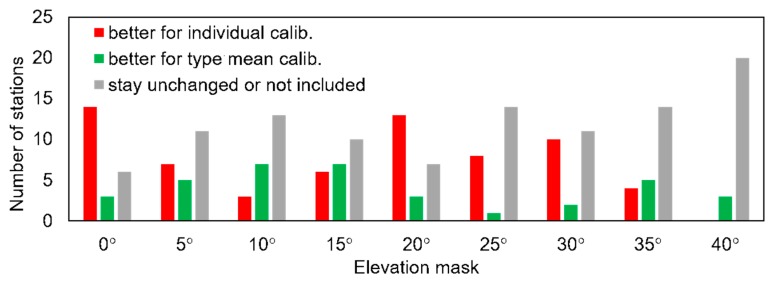
Height repeatability depending of applied elevation mask.

The main products of analysis were 18 coordinate time series per each station, two for each elevation mask. In this study, we focused only on the heights. Firstly, we checked the repeatability of the estimated heights in relation to the used elevation mask (Table A3 and Table A4, Appendix B). Then, we excluded from further analysis all values that exceeded more than three times the initial values, which came from the solution where no mask was applied. The results obtained for the tested PCC tables were very similar. For solutions where no mask was applied, the individual calibrations gave better results for 15 stations, worse results for three stations, and the same results for seven stations (Figure 3). This outcome changed for higher masks (5° and 10°), where we received better repeatability for type mean calibrations. For even higher masks, individual calibrations showed their advantage again. In general, the individual calibrations improved solutions for more cases. However, the improvements were very small at no more than 0.2 mm.

**Figure 4:**
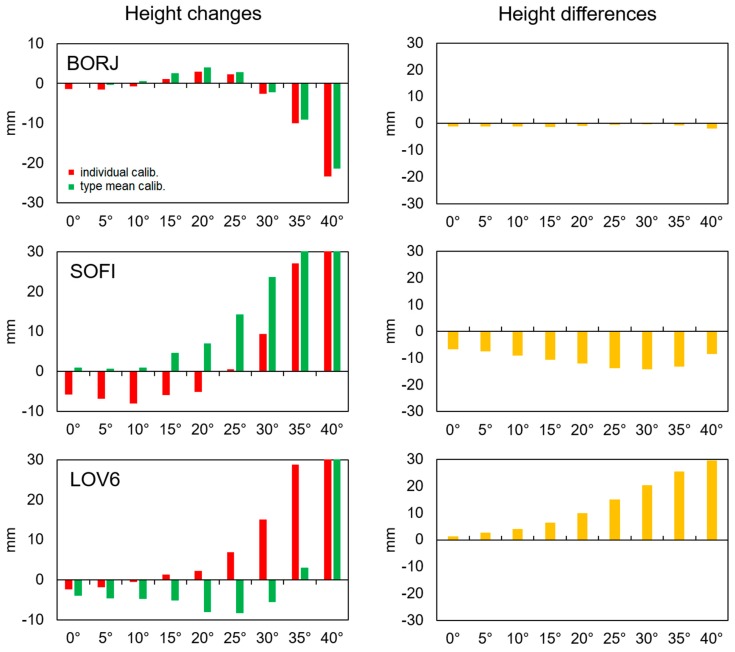
On the left, height changes related to applied elevation mask are shown. For each station, the reference height was calculated as a weighted average from all 18 solutions. On the right, height differences between corresponding solutions are shown (green − red).

The dependence between minimum observation angle and the estimated height uncertainty was quite obvious and came from the satellite geometry. Assuming that other factors like tropospheric delay are modeled the same way, we can interpret height differences between the solutions as the consequence of using PCCs. Therefore, we checked for which source of PCCs heights were more stable upon increasing the elevation mask (Table A5, Appendix C). We distinguished three groups of stations (Figure 4). The first one (e.g., BORJ, VIL6) showed quite good consistency between solutions regardless of the mask. The second group contained stations (e.g., SOFI) which showed a systematic bias between solutions independently of the used elevation mask. The last group contained stations (LEK7, LOV6) for which a higher mask also caused higher differences between corresponding solutions.

It should be mentioned here that the height of individual stations varied in different ways. This was a combined effect of many factors, such as the satellite geometry, environmental effects, and the used antenna. There were stations for which height decreased when a higher mask was adopted, as well as the opposite where height increased. There were also more complicated cases, where the estimated height firstly decreased and then increased. Changes generally did not exceed a single centimeter, even for high elevation angles.

## 4. Discussion

**Figure 5:**
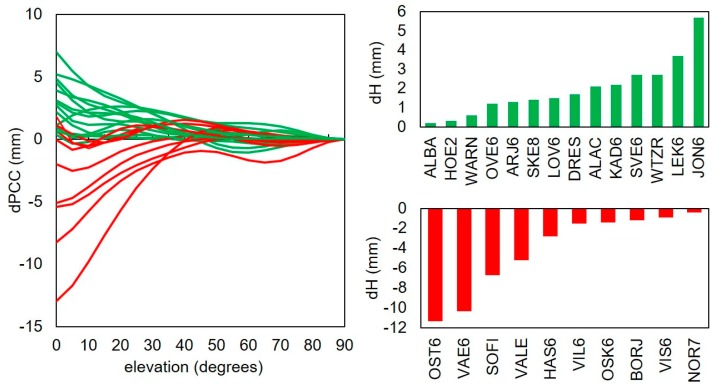
Comparison of the difference in PCC (dPCC; left) and mean differences between solution sol_i_0 and sol_t_0 (right). Stations with positive bias are shown in green, while those with negative bias are shown in red.

The obtained results showed that there were some differences for various PCC tables. There was a clear correlation between dPCC patterns and estimated differences in height. For each antenna where the dPCC exhibited a higher discrepancy at lower elevations, the height was more affected. When the dPCC exhibited a negative value at lower elevation, the height differences were also negative (e.g., OST6, SOFI). Of course, for smaller dPCC values and higher elevation masks, this relationship was not so clear. Figure 5 presents the dPCCs for all analyzed antennas, together with height differences for one pair of solutions.

**Figure 6:**
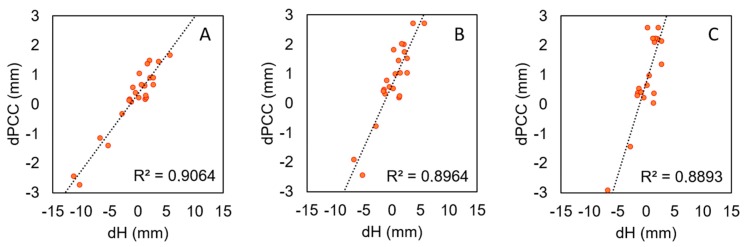
Correlation between mean value of dPCCs for selected sections and estimated height differences (dH). Three sections of dPCC are shown: (**A**) from 0 to 90; (**B**) from 0 to 50; (**C**) from 0 to 15.

Based on the analyzed dPCCs, a linear relationship could be shown between the mean dPCC values and the resulting differences in height. For various sections of dPCC, the Pearson’s correlation coefficient value was close to 0.9 (Figure 6). If the mean dPCC was negative, the height change was also negative. In particular, for antennas where dPCCs at lower elevation were significant (>2 mm), the mean value corresponded to the expected height difference. We compared the mean value of dPCCs with the height differences. The mean value of dPCCs for the lower section (below 10°) was close to height differences. The mean difference was 0.8 mm with an RMS of 1.4 mm. On this basis, one can try to estimate the expected difference in height resulting from the use of different models of phase centers. However, any conclusions should be drawn very carefully, because, for higher elevations, dPCCs are generally smaller and the dependence is not so clear. 

During the analysis, we checked which PCCs gave a more stable result. Two criteria were adopted for comparison. The first one referred to the height repeatability for particular variants of the applied mask. In our analysis, we showed that the repeatability does not depend on the used PCC source. For 17 stations, individual calibration gave better results, but the improvement should be considered as rather insignificant (see values in Appendix B). The second criterion was the height stability when the elevation mask was increased. In general, both sources of PCCs gave comparable results. For solutions from 0° to 25°, results were almost the same. The mean value of stability differences was 0.1 and varied from −2.7 mm to 2.8 mm. Therefore, one should conclude that the source of PCC has no effect on this. Including less accurate solutions (from 30° to 40°), the differences were bigger (up to 9 mm), but the number of more stable stations was still similar for individual (11) and type mean (12) calibrations.

**Figure 7:**
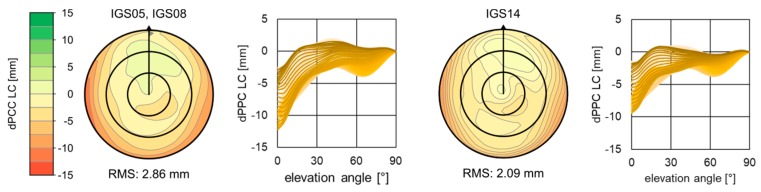
Difference in phase centre correction between individual model (#SN: 40012, mounted at OST6, Sweden) and International GNSS Service (IGS) type mean consecutive releases (IGS05, IGS08, and IGS14).

Most of the stations used in the analyses had available individual calibrations that were not included in the IGS08 models, especially the new stations in Sweden. For the selected antenna type, the IGS model was based only on 10 calibrations of five antennas. This is not a large sample, which may explain the received differences. For the current release, IGS14 [34], a type mean model for LEIAR25.R3 LEIT includes 54 calibrations of 28 antennas in total, as well as those installed at Swedish stations. Including more calibration makes IGS models more reliable. In our case, this did not change much. For antennas where negative bias was noted (Figure 5), the new release of the type mean model looked more consistent with individual calibration (Figure 7). However, for stations with a positive bias, we had the opposite effect. In general, all biases in height only changed by about +4 mm, which was already explained in IGSMAIL-7399 “Upcoming switch to IGS14/igs14.atx”. Therefore, based on the received biases (Table A5, Appendix C, and Figure 5) we can now conclude the impact of changing from type mean to individual models (or contrariwise). For OST6 we saw an improvement (Figure 7) and the height difference was about 8 mm (instead of 12 mm for igs08). However, for JON6 (Sweden), it was close to 10 mm.

## 5. Conclusions

This paper presents the impact of modelling the antenna phase centre on the estimated height. The comparison concerned two sets of phase center corrections used by the EUREF community: individual and type mean. During the analysis, we shown that for the most frequently used elevation masks (up to 10°), the consistency of dPCCs and the obtained height differences is high enough that the described method can be used for a first estimation of the impact of PCCs on the estimated heights.

Analyzes have shown that depending on the PCC source, the results may differ over 1 cm. However, this does not translate into repeatability of coordinates. It cannot be indicated which PCCs give significantly better results. The introduction of the new release of IGS type mean model did not change much. Only systematic differences have changed, which result from the differences between IGS08 and IGS14.

## Figures and Tables

**Table 1:** Solutions prepared in this study. PCC—phase center correction.

Name	Source of PCC	Elevation Mask	Name	Source of PCC	Elevation Mask
sol_t_00	igs_08.atx	0°	sol_i_00	epnc.atx	0°
sol_t_05		5°	sol_i_05		5°
sol_t_10		10°	sol_i_10		10°
sol_t_15		15°	sol_i_15		15°
sol_t_20		20°	sol_i_20		20°
sol_t_25		25°	sol_i_25		25°
sol_t_30		30°	sol_i_30		30°
sol_t_35		35°	sol_i_35		35°
sol_t_40		40°	sol_i_40		40°

## Appendix A

**Table A1:** Total RMSs of the differences between compared phase center corrections for both GPS frequencies (L1 and L2) and “ionosphere-free” linear combination (LC). Values are in millimeters.

Station	L1	L2	LC	Station	L1	L2	LC
ALAC	0.88	0.67	1.66	OST6	1.06	0.68	2.92
ALBA	0.39	0.65	1.70	OVE6	0.90	0.38	2.32
ARJ6	0.97	0.50	2.48	SKE8	0.49	0.89	1.66
BORJ	0.58	0.52	1.03	SOFI	0.83	0.24	2.36
DRES	0.61	1.36	1.42	SVE6	0.26	0.82	1.45
HAS6	0.63	0.73	1.42	VAE6	1.57	0.93	4.86
JON6	0.77	0.71	2.00	VALE	1.21	0.94	3.19
KAD6	0.72	0.39	2.20	VIL6	0.49	0.98	1.37
LEK6	1.16	0.45	2.46	VIS6	0.57	0.39	1.43
LOV6	0.71	0.37	2.14	WARN	0.54	0.53	1.40
NOR7	0.51	0.54	1.15	WTZR	0.74	0.90	1.47
OSK6	0.76	0.72	1.30				

**Table A2:** RMSs of the phase center correction differences for “ionosphere-free” linear combination depending on the elevation angle. Values are in millimeters.

Station	0–10°	15–25°	30–40°	45–55°	60–70°	75–85°
ALAC	1.83	1.94	1.65	1.16	0.69	0.28
ALBA	2.47	1.59	0.92	0.48	0.54	0.47
ARJ6	2.80	2.33	1.93	1.91	1.60	0.89
BORJ	1.72	1.14	0.72	0.46	0.44	0.23
DRES	1.35	0.71	0.55	0.53	0.28	0.25
HAS6	0.80	0.59	0.33	0.68	0.97	0.70
HOE2	1.44	0.58	0.39	0.51	0.61	0.36
JON6	1.00	0.75	0.57	0.79	0.59	0.14
KAD6	1.22	0.79	0.89	1.03	1.00	0.67
LEK6	1.37	1.04	0.82	0.73	0.70	0.43
LOV6	1.77	1.54	1.34	1.09	1.06	0.56
NOR7	1.67	1.02	0.64	0.71	0.72	0.44
OSK6	1.35	1.15	1.28	1.10	1.03	0.68
OST6	2.61	2.05	1.64	1.42	1.42	0.80
OVE6	2.12	1.78	1.49	1.24	0.69	0.36
SKE8	2.49	2.22	1.46	1.10	0.85	0.49
SOFI	1.88	1.35	0.98	0.64	0.59	0.36
SVE6	1.75	1.42	1.26	1.10	0.86	0.49
VAE6	2.97	2.76	2.15	1.59	0.90	0.45
VALE	2.44	2.66	2.57	2.13	1.34	0.53
VIL6	1.70	1.35	0.94	0.94	0.86	0.35
VIS6	1.91	1.36	0.74	0.66	0.62	0.40
WARN	1.69	1.38	1.51	1.20	0.80	0.38
WTZR	1.78	1.37	1.02	0.70	0.36	0.07
Mean	1.84	1.45	1.16	1.00	0.81	0.45

## Appendix B

**Table A3:** Standard deviations of height for solutions based on individual calibrations. Values are in millimeters.

Station	0°	5°	10°	15°	20°	25°	30°	35°	40°
ALAC	5.2	4.9	5.6	6.6	8.2	10.2	13.9	19.4	29.6
ALBA	5.5	5.4	6.0	6.8	8.5	10.7	13.4	16.8	23.7
ARJ6	8.0	8.7	9.8	11.0	13.0	17.9	24.3	31.4	45.7
BORJ	4.4	4.2	4.5	5.5	7.1	9.0	10.9	15.4	21.7
DRES	4.7	4.1	4.0	4.7	5.7	7.5	9.8	12.3	16.3
HAS6	5.2	5.3	5.5	6.0	6.5	7.4	9.4	12.5	16.5
HOE2	3.9	3.6	3.9	4.4	4.8	5.1	6.8	9.2	16.0
JON6	5.1	5.2	5.7	6.4	7.3	8.1	9.8	13.3	21.6
KAD6	6.0	6.1	6.3	6.8	7.7	9.2	10.8	13.9	20.1
LEK6	5.1	5.1	5.3	5.6	6.1	6.9	8.3	10.6	16.9
LOV6	5.7	5.7	5.7	5.8	6.3	7.3	8.8	11.8	14.9
NOR7	5.0	4.8	4.8	4.9	5.2	5.6	7.1	9.9	13.7
OSK6	5.2	5.1	5.2	5.4	5.8	6.4	7.7	10.0	14.8
OST6	6.1	5.7	5.7	6.2	6.8	8.2	10.1	13.0	20.9
OVE6	6.8	6.9	7.1	7.6	8.6	10.4	14.3	23.5	31.9
SKE8	5.7	5.7	5.7	5.8	5.9	6.2	7.6	10.8	17.9
SOFI	6.5	6.6	6.8	7.6	8.2	9.7	10.7	13.1	21.7
SVE6	6.4	6.5	7.4	9.2	11.3	15.0	20.0	24.9	31.9
VAE6	5.9	5.7	5.7	5.8	6.1	6.8	7.9	10.4	16.2
VALE	5.2	5.3	5.7	6.1	6.5	7.1	8.7	11.8	17.5
VIL6	4.9	4.6	5.1	6.1	7.7	9.5	13.0	16.1	22.5
VIS6	9.9	10.8	11.9	13.9	16.7	22.0	31.5	47.8	73.7
WARN	5.5	5.4	5.5	5.6	5.6	5.9	7.4	10.2	15.1
WTZR	4.8	4.2	4.1	4.6	5.2	5.8	7.6	9.9	14.1

**Table A4:** Standard deviations of height for solutions based on type mean calibrations. Values are in millimeters.

Station	0°	5°	10°	15°	20°	25°	30°	35°	40°
ALAC	5.3	4.8	5.4	6.5	8.2	10.2	13.8	19.2	29.0
ALBA	5.3	5.3	5.8	6.8	8.4	10.7	13.4	16.7	23.3
ARJ6	8.1	8.7	9.8	11.1	13.1	17.9	24.3	31.5	45.9
BORJ	4.4	4.1	4.5	5.4	7.0	9.0	11.1	15.6	21.3
DRES	4.7	4.1	4.1	4.6	5.7	7.5	9.8	12.2	15.8
HAS6	5.4	5.3	5.5	6.0	6.6	7.5	9.5	12.7	16.5
HOE2	4.0	3.5	3.9	4.4	4.7	5.1	6.6	9.2	15.9
JON6	5.3	5.3	5.7	6.5	7.4	8.1	9.7	13.3	21.9
KAD6	6.1	6.1	6.2	6.8	7.8	9.3	10.9	13.6	20.1
LEK6	5.1	5.2	5.4	5.7	6.2	6.9	8.4	10.5	16.7
LOV6	5.8	5.6	5.7	5.8	6.4	7.2	8.9	11.8	14.4
NOR7	5.1	4.9	4.7	5.0	5.3	5.7	7.1	10.2	13.7
OSK6	5.4	5.2	5.1	5.4	5.9	6.4	7.8	10.1	14.4
OST6	6.2	5.7	5.7	6.1	6.8	8.2	10.2	13.1	20.5
OVE6	6.9	7.0	7.1	7.8	8.9	10.6	14.5	23.6	32.3
SKE8	5.7	5.7	5.7	5.8	6.0	6.3	7.6	10.8	17.8
SOFI	6.7	6.6	6.8	7.7	8.3	9.7	10.7	13.1	21.7
SVE6	6.3	6.5	7.5	9.1	11.3	15.1	19.9	25.0	31.8
VAE6	5.9	5.7	5.6	5.8	6.2	6.8	7.9	10.3	16.2
VALE	5.4	5.4	5.7	6.1	6.5	7.1	8.7	11.7	17.3
VIL6	4.8	4.6	5.1	6.1	7.8	9.7	13.2	16.1	22.2
VIS6	9.9	10.7	11.9	13.8	16.7	22.1	31.9	48.1	73.3
WARN	5.6	5.5	5.5	5.6	5.7	6.0	7.4	10.2	14.9
WTZR	4.9	4.2	4.0	4.6	5.2	5.8	7.6	9.9	14.1

## Appendix C

**Table A5:** Biases in height between solutions sol_i_x and sol_t_xx, where xx is the applied mask. Values are in millimeters.

Station	0°	5°	10°	15°	20°	25°	30°	35°	40°	RMS
ALAC	2.1	2.1	1.8	1.6	1.8	2.2	1.7	−1.1	−7.0	2.8
ALBA	0.2	0.0	−0.5	−1.7	−2.6	−3.1	−3.4	−2.4	0.7	1.5
ARJ6	1.3	1.4	1.3	1.2	0.4	−1.8	−6.2	−12.6	−19.2	7.0
BORJ	−1.2	−1.2	−1.3	−1.4	−1.0	−0.6	−0.5	−0.9	−2.0	0.4
DRES	1.7	1.8	1.9	2.2	2.6	3.3	4.1	4.2	2.2	0.9
HAS6	−2.8	−3.4	−5.0	−6.5	−8.0	−9.9	−12.8	−16.5	−21.1	5.9
HOE2	0.3	0.5	0.5	0.0	−0.3	−0.6	-0.9	−1.2	−2.6	0.9
JON6	5.7	7.0	8.7	10.7	12.9	15.5	17.8	18.4	15.6	4.4
KAD6	2.2	2.9	3.2	3.6	4.6	5.6	6.6	6.5	5.5	1.5
LEK6	3.7	4.5	4.8	6.1	8.7	13.2	19.5	26.3	31.9	9.8
LOV6	1.5	2.8	4.2	6.5	10.2	15.2	20.5	25.7	30.9	10.0
NOR7	−0.4	−1.6	−4.0	−6.1	−7.5	−8.3	−8.0	−6.9	−6.0	2.7
OSK6	−1.4	−2.1	−3.8	−4.7	−4.8	−3.8	−2.9	−2.0	−0.5	1.4
OST6	−11.3	−13.0	−15.2	−17.6	−19.9	−22.0	−23.6	−23.5	−20.8	4.3
OVE6	1.2	1.6	1.5	2.1	4.8	9.8	16.7	25.5	34.2	11.4
SKE8	1.4	1.9	2.5	3.5	5.5	8.2	11.2	13.0	10.5	4.2
SOFI	−6.7	−7.5	−9.0	−10.6	−12.1	−13.8	−14.2	−13.2	−8.5	2.7
SVE6	2.7	3.0	3.2	3.1	3.0	2.0	−1.0	-5.8	−11.8	5.0
VAE6	−10.3	−13.2	−17.6	−21.6	−26.0	−30.8	−36.0	−40.3	−43.7	11.2
VALE	−5.2	−6.0	−7.2	−9.0	−10.5	−12.3	−13.9	−15.8	−17.0	4.0
VIL6	−1.5	−1.7	−2.2	−2.5	−1.9	−1.0	−0.6	−1.8	−5.5	1.3
VIS6	−0.9	−1.9	−3.7	−5.7	−7.1	−7.0	−5.4	−2.3	1.0	2.7
WARN	−0.6	−0.8	−1.4	−1.6	−0.9	0.9	1.9	2.3	1.2	1.4
WTZR	2.7	3.3	3.8	5.1	7.5	10.5	13.3	15.6	16.7	5.2
